# Effects of Long-Term Coated Sodium Butyrate Supplementation on the Intestinal Health and Colonization of Cecal *Salmonella* of Laying Hens Infected with *Salmonella enteritidis*

**DOI:** 10.3390/ani14091356

**Published:** 2024-04-30

**Authors:** Siyu Xiong, Qi Zhang, Keying Zhang, Jianping Wang, Shiping Bai, Qiufeng Zeng, Huanwei Peng, Yue Xuan, Yadong Mu, Xuemei Ding

**Affiliations:** Institute of Animal Nutrition, Key Laboratory for Animal Disease-Resistance Nutrition of China Ministry of Education, Sichuan Agricultural University, 211 Huimin Road, Wenjiang District, Chengdu 611130, China

**Keywords:** coated sodium butyrate, *Salmonella enteritidis*, laying hens, intestinal health

## Abstract

**Simple Summary:**

*Salmonella enterica* ser. Enteritidis (*S. Enteritidis*) is widely found in chickens and eggs and has the potential to induce human illness. Infection with *Salmonella enteritidis* triggers inflammatory responses in the gut and body, leading to decreased production performance of poultry. Sodium butyrate (SB) is an alternative antibiotic used as an acidifier that can be transformed into butyric acid (BA) in the avian alimentary canal. BA, a short-chain fatty acid, is often used extensively in poultry production against *Salmonella.* Since an unpleasant odor can affect feed intake, coated sodium butyrate (CSB) is more widely used. However, the effects of CSB on intestinal health in hens infected with *S. Enteritidis* have been less reported. Therefore, this study aimed to investigate the effects of long-term dietary supplementation with CSB on the intestinal health and colonization of cecum *Salmonella* from laying hens infected with *Salmonella enteritidis.* The obtained results revealed that long-term dietary CSB improved the gut morphology and alleviated intestinal injury and the colonization of cecum *Salmonella* of laying hens infected with *S. Enteritidis*. Moreover, long-term supplementation of CSB in laying hens resulted in a better intestinal barrier, which effectively protected the intestinal health of laying hens and reduced *Salmonella* challenges.

**Abstract:**

*Salmonella enterica* ser. Enteritidis (*S. Enteritidis*) is widely found in chickens and eggs, and it can potentially induce human illness. The investigation in this study centers on the impacts of long-term dietary supplementation with coated sodium butyrate (CSB) on intestinal well-being and the colonization of cecum *Salmonella* in laying hens infected with *S. Enteritidis.* We segregated a total of 120 Lohmann laying hens aged 51 weeks into four treatment categories: 0 (CON), 300 (CSB1), 500 (CSB2), and 800 (CSB3) mg/kg of CSB, supplemented with CSB from the first day of the experiment. A 24-week observation process was carried out for each laying hen. The *S. Enteritidis* was orally administered to all chickens on the morning of the first and third days of week 22 of the trial. After the *S. Enteritidis* challenge, egg production decreased the most in the CON group. Compared to the CON group, the three doses of CSB significantly improved egg production after the *S. Enteritidis* challenge (*P_ANOVA_* < 0.05). *S. Enteritidis* challenge increased plasma DAO activity, but CSB supplementation reduced plasma DAO activity (*P_line__ar_* < 0.05). The *S. Enteritidis* challenge disrupted intestinal villi morphology; compared to the CON group, the three dosages of CSB resulted in an increase in villus height (VH) and the ratio of villus height to crypt depth (V/C) in the duodenum, jejunum, and ileum of infected laying hens (*P_line__ar_* < 0.05), with a significant increase in jejunal villus height (*P_ANOVA_* < 0.05). A decrease in ileal crypt depth was also observed (*P_line__ar_* < 0.05). CSB2 and CSB3 markedly increased the content of butyric acid in the cecum (*P_ANOVA_* < 0.05). Additionally, in contrast to those in the CON group, the propionic acid content in the CSB supplementation group increased (*P_line__ar_* < 0.05). Compared with those in the CON group, mRNA relative expression of the *IL-6* and *IL-1β* in jejunum (*P_line__ar_* < 0.05) and mRNA relative expression of the *IL-1β* in ileum (*P_ANOVA_* < 0.05) were significantly lower, and mRNA relative expression of the *IL-10* in ileum (*P_line__ar_* < 0.05) were significantly higher in the CSB group. In addition, in contrast to the CON group, the CSB supplementation group significantly upregulated mRNA relative expression of the *ZO-1* and *CLDN1* (*P_ANOVA_* < 0.05). Additionally, CSB supplementation reduced the number of *Salmonella* and increased the number of Lactobacilli in the cecum (*P_line__ar_* < 0.05) and tended to increase the total bacteria count (*P_line__ar_* = 0.069) and reduce the *E. coli* count (*P_line__ar_* = 0.081). In conclusion, long-term dietary supplementation with coated sodium butyrate can alleviate intestinal injury and the colonization of cecum *Salmonella* in laying hens infected with *S. Enteritidis*.

## 1. Introduction

Eggs serve as an economical source of animal protein, and the Chinese poultry sector has achieved the highest global rankings in both total egg production and per capita [1]. In recent times, the poultry sector has introduced the “laying cycle extension plan” for laying hens. This plan entails prolonging the laying hens’ elimination age from 72 weeks to a range of 80 to 100 weeks, with the aim of reaching the target of “500 eggs produced at 100 weeks of age” [2]. However, with increasing age, laying hens experience severe metabolic disorders, decreased performance, decreased egg quality, and gradually weakened resistance after the peak laying period, which is more likely to cause disease [3].

*S. Enteritidis* is the leading cause of human foodborne challenges, and eggs are one of the important vectors for human challenges with *Salmonella* [1,4]. Infection with *Salmonella enteritidis* triggers inflammatory responses in the gut and body, leading to decreased production performance. Studies have demonstrated that *S. Enteritidis* damages the structure and function of epithelial tight junction proteins and alters paracellular permeability [5]. Adding feeding antibiotics and improving the environment of livestock houses are important measures taken to alleviate *Salmonella enteritidis* infection. Approximately 73% of the world’s antibiotics are used for the prevention and treatment of animal diseases; however, long-term exposure to antibiotics can easily cause bacterial resistance, and antibiotic abuse can damage gastrointestinal barrier function, destroy the structure of the microbiota, and lead to intestinal homeostasis imbalance [6]. As a consequence, viable, nonantibiotic alternatives are needed for the prevention and treatment of *S. Enteritidis.*

Sodium butyrate (SB) is an alternative antibiotic used as an acidifier that can be transformed into butyric acid in the avian alimentary canal and has generated much attention [7]. It has been suggested that SB can supply energy to intestinal epithelial cells; regulate immune, antibacterial, and anti-inflammatory effects; protect intestinal barrier structure and function; and improve intestinal morphology [7,8,9,10,11,12]. Additionally, butyric acid released by sodium butyrate in the digestive tract is often used extensively in poultry production as a method against *Salmonella* [4]. It has been proposed that BA may reduce the expression of virulence genes and the invasion of *Salmonella* in epithelial cells in vitro [13]. Multiple investigations in the poultry field have indicated that supplementation with BA can lead to a reduction in *Salmonella* colonization and shedding [14]. Since an unpleasant odor can affect feed intake, SB is often prepared in a variety of forms [15]. For animal feed, sodium butyrate is typically used in two forms: coated sodium butyrate (CSB) and uncoated sodium butyrate (UCSB). Studies have revealed that CSB is more effective at preventing the colonization of the cecum by *Salmonella* [15]. This is because fat is not easily digested or absorbed in the stomach. After CSB enters the intestine, the enveloping fat layer is gradually decomposed by the decomposition of related enzymes so that BA is slowly released throughout the entire intestine and fully absorbed by the intestinal epithelium to nourish the intestine [16].

In recent years, SB has been used in a number of experimental studies on poultry and livestock. However, the effects of long-term dietary supplementation with coated sodium butyrate on intestinal injury caused by *S. Enteritidis* and colonization of cecal *Salmonella* have been less reported. Therefore, this study aimed to investigate the effects of long-term dietary supplementation with coated sodium butyrate (CSB) on the intestinal health and colonization of cecal *Salmonella* of laying hens infected with *S. Enteritidis*.

## 2. *Materials and Methods*

The study was approved by the Animal Care and Use Committee, Sichuan Agricultural University (Ethic Approval Code: SICAUAC202110-2; Chengdu, China).

### 2.1. Birds, Diet, and Management

Approval for the experimental protocols used in the study was provided by the Animal Care and Use Committee of Sichuan Agricultural University, China. In total, 120 Lohmann laying hens were adaptively pre-fed a control diet for 2 weeks. The hens with similar egg production rates and aged 51 to 74 weeks were divided into four treatment groups: 0 (CON), 300 (CSB1), 500 (CSB2), and 800 (CSB3) mg/kg CSB. For each treatment, five replicates were employed, with each chicken assigned to a single cage. A group of six consecutive cages formed one replicate. The CSB utilized in this investigation was purchased from Adisseo Life Sciences Products (Shanghai) Co., Ltd., Shanghai, China. Nipple-type drinkers and a feed trough were provided along the length of each cage. The birds were reared in an environment under controlled conditions, maintaining a temperature of 24 °C, humidity levels between 50 and 65%, and a 16 h light–dark cycle. Throughout the 24-week experimental period, all birds had unrestricted access to both water and the experimental diets. The mental state of the hens was observed daily, and mortality was recorded promptly. The baseline diet was a corn–soybean diet, with nutrient content and levels established using the National Research Council (NRC) (1994) and the Chinese Chicken Breeding Standard (2004) (Table 1).

### 2.2. Salmonella enteritidis Challenge Test

*Salmonella enteritidis* (CVCC3374) was acquired from Sichuan Agricultural University. Without eliminating the initial *Salmonella* interference agent, a 1 mL inoculation containing 1 × 10^8^ CFU/mL of *Salmonella enteritidis* was orally administered to all the chickens on the mornings of the first and third days of week 22 of the trial. Our previous study had effectively established an *S. Enteritidis* challenge model.

### 2.3. Sample Collection and Determination

Daily egg collection was performed, and both the total egg weight and the quantity of eggs in each replicate were documented. Mortality data were recorded daily throughout the duration of the experiment. At the end of week 24 of the trial, 20 hens were randomly chosen (1 from each replicate), and a 6 mL blood sample was extracted from the jugular vein and placed into two anticoagulant tubes containing EDTA-K2. These tubes were gently agitated to ensure thorough mixing of the blood and anticoagulant. Subsequently, all tubes were positioned at an angle in a 37 °C environment for 30 min and then centrifuged at 3000× *g* for 10 min at 4 °C to isolate plasma, which was stored at −20 °C for subsequent analyses. After blood collection, all the hens were sacrificed via cervical dislocation. Approximately 2 cm long duodenal, jejunal, and ileal segments were then removed and preserved in 4% neutral formaldehyde for histological analysis [7]. In order to explore short-chain fatty acids, fresh cecal contents were further extracted, put into sterile microtubes, and preserved at −80 °C. [17].

### 2.4. Intestinal Morphology

The intestinal segments were sectioned using a Leica CM1860 microtome after being soaked in 4% paraformaldehyde, dehydrated with ethanol, cleaned with xylene, and embedded in paraffin wax; the tissues were then cut into 5 mm thin sections, which were then placed on glass slides and stained with hematoxylin–eosin [18], followed by the determination of crypt depth (CD) and villus height (VH). From each sample, ten straight, intact villi were chosen, and Image-Pro Plus 6.0 (Media Cybernetics, Inc., Bethesda, MD, USA) was used to examine their morphology. The CD denotes the depth of invagination between adjacent villi, while the distance between the villus top and the crypt–villus junction is indicated by the VH. The VH/CD was defined as the ratio of VH to CD.

### 2.5. Intestinal Permeability

For the plasma samples (10 µL), sample diluent (40 µL) and HPR-conjugate reagents (100 µL) were added. After closing the plate with a closure plate membrane, it was incubated for 30 min at 37 °C. Subsequently, the liquid was discarded, and the plate was dried using a swinging motion. A washing buffer was added to every well, allowed to stand for 1 min, and then drained. This process was repeated 5 times, and the plate was dried by patting. Next, Chromogen Solution A (50 µL) and Chromogen Solution B (50 µL) were added to each well, the samples were incubated in the dark for 15 min at 37 °C, and stop solution (50 µL) was added to each well. The reaction was stopped, and the absorbance was subsequently read at 450 nm. Commercial enzyme-linked immunosorbent assay (ELISA) kits were purchased from Jiangsu Enzyme-linked Immunoassay Industry Co., Nanjing, China.

### 2.6. Expression of Intestinal Inflammatory Factors and Barrier Function Related Genes

The jejunum and ileum mucosa were used to extract total RNA using the TRIzol reagent (TaKaRa, Dalian, China), and reverse transcription was used to create cDNA. The quantitative real-time PCR was determined by an ABI Prism 7000 detection system with a 2-step protocol and SYBR Green (TaKaRa, Dalian, China). The primer sequences for all the genes (*OCLN*, *ZO-1*, *MUC2*, *IL-10*, *IL-1β*, *IL-6*, and *IFN-γ*) are listed in Table 2. Using the 2-ΔΔCT method to calculate the gene expression, the standard curve was generated by the tenfold gradient dilution method, with three replicates for each sample. Three replicates of each sample were analyzed, and β-actin was chosen as the housekeeping gene. The sequences of primers used were synthesized by Chengdu Bioengineering Co., Chengdu, China.

### 2.7. SCFA Concentrations

The concentrations of acetic acid, propionic acid, isobutyric acid, butyric acid, isovaleric acid, valeric acid, and total short-chain fatty acid in the cecal chyme were determined using a gas chromatograph (VARIAN CP-3800, Agelent Technologies Inc., Santa Clara, CA, USA). About 0.7 g of the sample (with its mass accurately recorded) was placed into a 2 mL centrifuge tube. This was followed by dilution with 1.5 mL of ultrapure water. The mixture was allowed to stand for 30 min and then centrifuged at 20,000× *g* for 15 min to obtain the extract, with a sample concentration that was denoted as M. Subsequently, 1 mL of the supernatant was transferred to a new tube and mixed with 23.3 mL of 210 mmol/L crotonic acid and 0.2 mL of 25% metaphosphoric acid. After incubating the mixture for 30 min at 4 °C, it was centrifuged at 20,000× *g* for 10 min, followed by filtration into a 1.5 mL tube. Methanol (0.9 mL) was subsequently added, and the mixture was subjected to 5 min of centrifugation at 10,000× *g*, followed by filtration of the supernatant with a 0.22 mm membrane and collection in 1.5 mL tubes for further analysis [19].

### 2.8. Cecal Microbial Analysis

The cecum was isolated, and cecal chyme was collected, with subsequent storage of the samples in liquid nitrogen. The total intestinal microorganisms, including total bacteria, Lactobacillus, *E. coli*, and *Salmonella*, were quantified. The DNA extraction kit was procured from Bioengineering (Shanghai) Co., Shanghai, China. RT–PCR was employed to quantify microorganism numbers in the cecum, utilizing a Taqman probe. The kit was acquired from Tiangen Biochemical Technology (Beijing) Co., Beijing, China. The common logarithm of the number of bacteria per gram of content [log (copies/g)] is used to express the results. The sequences of primers used are provided in Table 3.

### 2.9. Statistical Analysis

Statistical analysis was conducted using one-way ANOVA with the SAS 9.2 general linear model (GLM) package (version 9.2; SAS Institute, Inc., Cary, NC, USA). When a significant therapeutic effect was observed among multiple comparisons, Duncan’s test was employed to compare the means of various treatments. The impact of the dosage of CSB supplementation on various parameters was assessed using the contrast statement in both linear and quadratic trend analysis. Furthermore, broken-line, asymptotic, and quadratic model regressions were carried out using the nonlinear (NLIN) procedure of SAS, and the best-fit model was chosen according to the coefficient of determination and *p*-value [20]. The results are displayed as the means and standard errors of the means, where *p* < 0.05 indicates statistical significance and 0.05 ≤ *p* < 0.1 indicates a statistically significant trend.

## 3. Results

### 3.1. Production Performance

During the *S. Enteritidis* challenge, a total of four chickens perished in the CON group, two chickens in the CSB1 group, one chicken in the CSB2 group, and no chicken fatalities were recorded in the CSB3 group. On the third day following the challenge, Pullorum infection was observed in the chickens, and on the morning of the fourth day, green feces were noticed in the CON group. By day 5, slight green feces were observed in a few chickens in the CSB treatment. On day 6, the CON group exhibited significant amounts of green feces. After *Salmonella enteritidis* challenge, the survival rate was 86.67% in the CON group, 93.33% in the CSB1 group, 96.67% in the CSB2 group, and 100% in the CSB3 group. After the *Salmonella enteritidis* challenge, egg production decreased the most in the CON group. Long-term CSB supplementation prevented the decrease in egg production induced by *S. Enteritidis*. Compared to the CON group, the three doses of CSB significantly improved egg production after the *S. Enteritidis* challenge (*P_ANOVA_* < 0.05) (Table 4).

### 3.2. Intestinal Morphology

The *S. Enteritidis* challenge disrupted intestinal morphology. Compared to the CON group, the three doses of CSB resulted in an increase in villus height and the ratio of villus height to crypt depth (V/C) in the duodenum, jejunum, and ileum of infected laying hens (*P_line__ar_* < 0.05) (Table 5), with a significant increase in jejunal villus height (*P_AVOVA_* < 0.05). A decrease in ileal crypt depth was also observed (*P_line__ar_* < 0.05).

### 3.3. Intestinal Permeability

**Figure 1:**
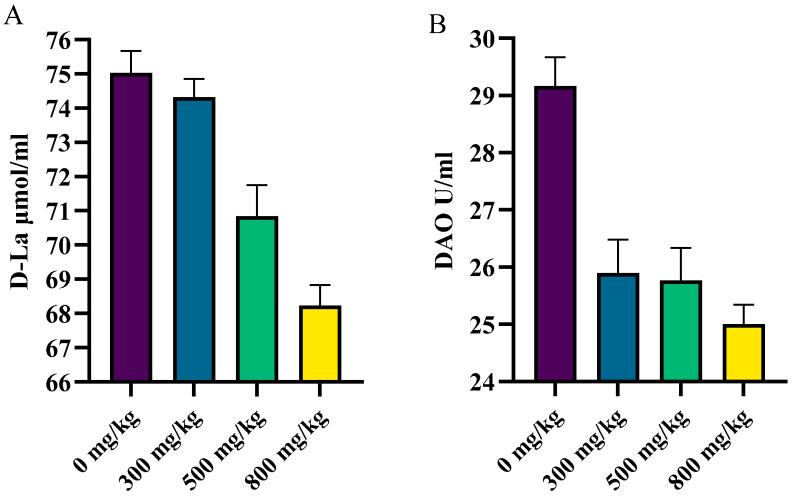
The effects of coated sodium butyrate on intestinal permeability of laying hens challenged with *Salmonella.* (**A**) The serum D-La content of laying hens was measured using the ELISA Kit. (**B**) The serum DAO content of laying hens was measured using the ELISA Kit. Data are means ± SEM.

The plasma DAO activity decreased linearly with increasing CSB (*P_line__ar_* < 0.05), but there was no significant difference in the plasma D-LA concentration (*P_ANOVA_* > 0.05) (Figure 1).

### 3.4. Expression of Intestinal Inflammatory Factors and Barrier Function-Related Genes

The expression level of *IL-1β* in the jejunum decreased linearly with increasing CSB (*P_line__ar_* < 0.05), and the expression level of *IL-6* in the jejunum also decreased linearly (*P_line__ar_* < 0.05) (Table 6). There were no significant differences in the relative expression of *IL-10* or *IFN-γ* in the jejunum between the CON and CSB groups (*P_ANOVA_* > 0.05). The expression level of *IL-1β* in the ileum was significantly higher in the CON group than in the other groups (*P_ANOVA_* < 0.05). The expression level of *IL-10* in the ileum increased linearly with increasing CSB (*P_line__ar_* < 0.05), while there was no significant difference in the expression of IL-6 or *IFN-γ* among the groups (*P_ANOVA_* > 0.05).

The expression of *ZO-1* in the jejunum significantly increased in the CSB3 group (*P_ANOVA_* < 0.05), and the expression level of *CLDN1* increased linearly with increasing levels of CSB (*P_line__ar_* < 0.05). The expression of MUC2 in the jejunum showed an increasing trend with increasing levels of CSB (*P_ANOVA_* = 0.084) (Table 7). In the ileum, the expression levels of *ZO-1* and *CLDN1* in the CSB groups were significantly greater than those in the CON group (*P*_ANOVA_ < 0.05).

### 3.5. SCFA Concentrations

The addition of CSB significantly increased the concentration of butyric acid (*P*_ANOVA_ < 0.05) and linearly increased the concentration of propionic acid in the cecal chyme after *Salmonella enteritidis* challenge (*P_linear_* < 0.05) (Table 8). Similarly, the concentrations of acetic acid (*P_linear_* = 0.079), valeric acid (*P_linear_* = 0.057), and total short-chain fatty acids (*P_linear_* = 0.060) increased linearly.

### 3.6. Cecal Microbial Analysis

**Figure 2:**
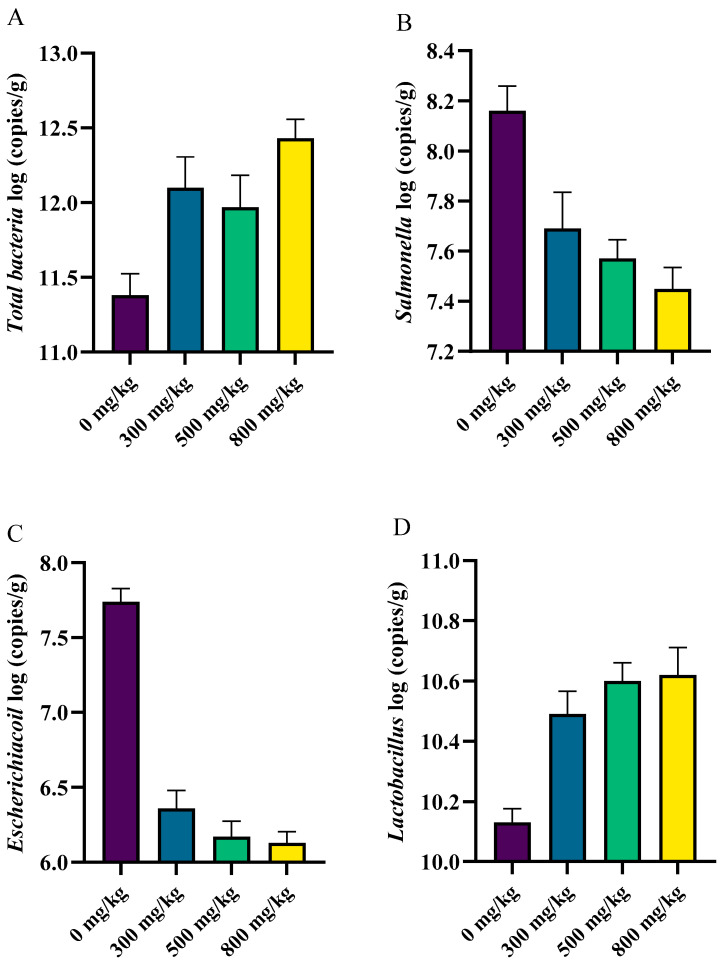
The effects of coated sodium butyrate on the cecal microbes of laying hens challenged with *Salmonella* (log (copies/g)). (**A**) Total bacteria in the cecum. (**B**) The count of *Salmonella* in the cecum. (**C**) The count of *E. coli* in the cecum. (**D**)The count of *Lactobacillus* in the cecum. Data are means ± SEM.

The *Salmonella* level decreased linearly with increasing levels of CSB after the *Salmonella enteritidis* challenge (*P_line__ar_* < 0.05), while the *Lactobacillus* level increased linearly (*P_line__ar_* < 0.05) (Figure 2). The *total bacteria* concentration showed a linear increasing trend (*P_line__ar_* = 0.069), and the *Escherichia coli* concentration showed a linear decreasing trend (*P_line__ar_* = 0.081).

## 4. Discussion

Infection with *S. Enteritidis* triggers inflammatory responses in the gut and body, leading to decreased production performance of poultry. In this study, we found that after the *S. Enteritidis* challenge, chicken deaths occurred, but fewer chickens died in the CSB groups than in the CON group, and no chickens died in the CSB3 group. After the challenge test, the egg production was significantly reduced, but compared to the CON group, the three doses of CSB significantly prevented the decrease in egg production induced by *S. Enteritidis*. SB has been reported to reduce the infection of *Salmonella enteritidis* in birds [21]. It has been reported that SB can bind to pathogens and stimulate the immune system. It also enhances intestinal function by improving villus uniformity. Furthermore, the enhanced production performance may be attributed to butyric acid produced by CSB, which exhibits potent bactericidal activity when in its undissociated form. Butyric acid can exert an antibacterial effect by penetrating the cell membrane of *Salmonella* and altering pH, ultimately leading to bacterial death [22].

In poultry, parameters like intestinal integrity are commonly used to evaluate the effectiveness of dietary additives. Song et al. [23] demonstrated that the inclusion of microencapsulated sodium butyrate in broiler diets prevented the reduction in villus height and V/C in the small intestine induced by necrotic enteritis. Moreover, the addition of CSB to the diet significantly increased duodenal villus height and V/C in the ileum while also significantly reducing crypt depth in the ileum in LPS-challenged broilers [24]. Similarly, in our study, the addition of CSB increased both duodenal and jejunal villus height and V/C after the *SE* challenge. In addition, various degrees of improvement in ileal villus height, crypt depth, and V/C were observed. These results could be due to the metabolite of CSB, butyric acid. BA can be used as a fast energy source for intestinal mucosal epithelial cells and can stimulate the growth of villi and maintain the normal morphology of the intestinal mucosa [25].

Intestinal permeability reflects the integrity of the intestinal barrier. Damage to the intestinal barrier leads to an increase in intestinal permeability, which causes D-lactic acid (D-LA) and diamine oxidase (DAO) to pass through the intestinal mucosa into the blood, resulting in increased D-LA and DAO levels; therefore, D-LA content and DAO activity can be used as indicators of intestinal permeability in poultry [26]. Zou et al. [27] reported that the addition of SB to the feed of broilers significantly suppressed the increase in serum D-LA levels induced by intestinal inflammation induced by dextran sulfate sodium. As demonstrated by the present study, after the *S. Enteritidis* challenge, the intestinal permeability in the CSB group was lower than that in the CON group, which is consistent with the findings of previous studies. The role of SB in maintaining the integrity of the intestinal barrier may be achieved by binding to GPRs, which activate signaling pathways regulating the immune response, as suggested by Li et al. [28]. Additionally, SB might regulate the expression of small intestinal tight junction proteins and inflammatory cytokines, thereby reducing *S. Enteritidis*-induced damage to the intestinal mucosal barrier.

Intestinal tight junction proteins play a crucial role in determining intestinal permeability and barrier function. Studies have shown that *Salmonella* can downregulate the expression of tight junction proteins such as ZO-1 and Claudin, leading to the disruption of tight junction architecture [5]. Feng et al. [29] reported that supplementing the diet of weaned piglets with CSB could upregulate the expression of claudin-3 and Occludin in the ileum. Elevated expression levels of mRNAs related to tight junction proteins, including Claudin-3, Occludin, and ZO-1, were also observed in the colon. Similarly, reported that butyrate significantly increased the expression of Occludin, Claudin-1, and ZO-1 in a rat model of severe acute pancreatitis with intra-abdominal hypertension. These findings align with the results of our own study, where dietary supplementation of CSB led to an upregulation of CLDN1, ZO-1, and MUC2 expression in laying hens following *S. Enteritidis* challenge [30]. Consequently, it can be hypothesized that CSB exerts a protective effect on the intestinal barrier of laying hens after exposure to *S. Enteritidis.*

Reducing the occurrence of intestinal inflammation can improve the immunity of animals, reduce intestinal permeability, and improve the function of the intestinal immune barrier [31]. Previous studies have shown that the *S. Enteritidis* challenge increases the relative expression of *IFN*-*γ* and *IL-6* mRNAs in the ileum of laying hens [32]. As observed in the present study, dietary CSB supplementation decreased the expression of *IL-6* and *IL*-*1β* mRNAs in the jejunum of laying hens after *S. Enteritidis* challenge and modulated the expression of inflammatory factors in the ileum, similar to previous findings [33]. Our previous investigations have demonstrated that supplementing laying hens’ diets results in a downregulation of *IL*-*1β* mRNA expression and an upregulation of *IL*-*10* mRNA expression in the jejunum. *IL-10* is a crucial anti-inflammatory cytokine. When CSB is metabolized into butyric acid (BA) in the intestine, BA functions as an inhibitor of histone deacetylases (iHDACs) and stimulates the production of *IL*-*10* by promoting the differentiation of regulatory T cells (Tregs). Subsequently, *IL*-*10* exerts its immunomodulatory effects by inhibiting the activation of T helper cells (Th cells) and suppressing the expression of *IL*-*1β* and *IL*-*6* mRNAs [34,35]. Consequently, BA, as a potent component of CSB, may enhance the resistance of laying hens to *Salmonella* by participating in the regulation of the humoral immune response mediated by Th2 cells in the intestinal mucosa.

A large number of microbes determine the health of the gut. SCFAs are major products of intestinal bacterial metabolism, and they play a crucial role in preventing the colonization of pathogens, including *Salmonella*, in the digestive tract [36]. Our study showed that dietary CSB supplementation increased the count of *Lactobacillus* and the levels of butyric acid, propionic acid, valeric acid, and total short-chain fatty acids in the cecum. Previous studies have also indicated that the inclusion of both SB and CSB in the diet results in elevated levels of SCFAs, particularly BAs [16]. Similarly, supplementing the diet with CSB significantly reduced the number of *Salmonella* in the cecum of poultry [14]. BA has been demonstrated to have good antimicrobial efficacy and has been widely used in broilers to control *Salmonella* infection [14]. In vitro, BA attenuated *Salmonella* invasion of intestinal epithelial cells. CSB has the capacity to modulate the composition of the intestinal microbiota by promoting the proliferation of beneficial bacteria and reducing the abundance of harmful bacteria, primarily through its principal metabolite, BA [4]. This, in turn, contributes to the preservation of microbial barrier function.

## 5. Conclusions

In conclusion, long-term dietary CSB supplementation improved the gut morphology of laying hens after the *S. Enteritidis* challenge. Moreover, long-term supplementation of CSB in laying hens resulted in a better intestinal barrier, which effectively protected the intestinal health of laying hens and reduced *Salmonella* challenges.

## Figures and Tables

**Table 1:** Composition and nutrient level of the basal diet (as-fed basis).

Ingredients	Contents, %
Corn (7.8% of CP)	62.39
Soybean meal (43% of CP)	19.48
Soybean oil	1.00
Corn protein meal (55% of CP)	3.60
Limestone (fine)	6.20
Limestone (coarse)	3.10
Dicalcium phosphate·2H_2_O	1.56
Lysine·H_2_SO_4_ (70%)	0.08
DL-Methionine (99%)	0.08
Unite bran	1.46
Sodium chloride	0.30
Choline chloride	0.10
Vitamin premix ^1^	0.50
Mineral premix ^2^	0.15
Total	100.00
Nutrient level, %	
ME, kcal/kg	2680.00
Crude protein	15.38
Calcium	4.00
Total phosphorus	0.57
Available phosphorus	0.37
D-Lysine	0.73
D-Methionine	0.34
D-Threonine	0.58
D-Tryptophan	0.16
D-Methionine + D-Cysteine	0.58

^1^ Vitamin premix provided the contents below in diet (/kg): VA, 9950 IU, VB1, 37.7 mg, VB2, 12 mg, D-pantothenate, 18.2 mg, VB6, 7.55 mg, VB12, 0.5 mg, VD3 5000 IU, VE:70 IU, VK3, 4.47 mg, Biotin, 4 mg, VC,195 mg, niacin acid, 70.35 mg; ^2^ Mineral Premix offered the contents below in diet (/kg): Cu (as copper sulfate), 9.6 mg; Fe (as ferrous sulfate), 64 mg; Mn (as manganese sulfate),121.5 mg; Zn (as zinc sulfate), 57 mg; I (as potassium iodide), 0.60 mg; Se (as sodium selenite), 0.36 mg.

**Table 2:** Sequences of the oligonucleotide primers used for quantitative real-time PCR.

Target Gene	Primer Sequences	Fragment (bp)	Gen Bank No.
*β-actin*	F: GCTACAGCTTCACCACCACA	90	NM_205518.2
	R: TCTCCTGCTCGAAATCCAGT		
*CLDN1*	F: GTCTTTGGTGGCGTGATCTT	117	NM_001013611.2
	R: TCTGGTGTTAACGGGTGTGA		
*ZO-1*	F: GAGCGCAAGTTTGAAAGTCC	135	XM_040680632.1
	R: AGGAGGCTGTGATGAGCTGT		
*MUC2*	F: TGCCAGCCTTTTTATGCTCT	80	XM_040673077.1
	R: AGTGGCCATGGTTTCTTGTC		
*IL-1β*	F: TCCTGGAGGAGGTTTTTGAG	128	NM_204524.2
	R: CACGAAGCACTTCTGGTTGA		
*IL-6*	F: CAGGACGAGATGTGCAAGAA	233	NM_204628.2
	R: TAGCACAGAGACTCGACGTT		
*IL-10*	F: CAATCCAGGGACGATGAACT	114	NM_001004414.3
	R: ATCTGTGTAGAAGCGCAGCA		
*IFN-γ*	F: AACAACCTTCCTGATGGCGT	106	NM_205149.2
	R: TGAAGAGTTCATTCGCGGCT		

Primers designed using Primer Express software 3.0 (Sangon Biotech, Shanghai, China); CLDN1 = Claudin-1; ZO-1 = zonula occludens-1; MUC2 = Mucin-2; F = forward; R = reverse.

**Table 3:** The specific 16S rRNA primers applied for quantifying bacteria in cecal content.

Items	Primer	Primer/Probe Sequence (5′-3′)	Amplicon Length
*Salmonella*	Forward	AGGCCTTCGGGTTGTAAAGT	20
	Reverse	GTTAGCCGGTGCTTCTTCTG	20
	Probe	AACCGCAGCAATTGACGTTACCC	23
*Lactobacillus*	Forward	GTGTCGTGAGATGTTGGGTTAAGTC	25
	Reverse	CCACCTTCCTCCGGTTTGT	19
	Probe	CGCAACCCTTATTATCAGTTGCCAGCA	27
*Escherichia coli*	Forward	AATGGCGCATACAAAGAGAAGC	22
	Reverse	GTTGCAGACTCCAATCCGGA	20
	Probe	CACTTTATGAGGTCCGCTTGCTCTCGC	27
*Total bacteria*	Forward	ACTCCTACGGGAGGCAGCAG	20
	Reverse	ATTACCGCGGCTGCTGG	17

**Table 4:** Effects of coated sodium butyrate on the performance of laying hens challenged with *Salmonella.*

Item	CSB Level (mg/kg)				SEM	*p*-Value		
	0	300	500	800		ANOVA	Linear	Quadratic
Egg production (%)								
one week before infected	55.71	56.67	56.67	57.14	0.32	0.538	0.183	0.761
after infected								
1–7 day	46.76 ^c^	50.29 ^b^	51.24 ^b^	54.29 ^a^	0.68	<0.01	<0.01	0.702
8–14 day	36.57 ^c^	42.29 ^b^	49.33 ^a^	50.95 ^a^	1.36	<0.01	<0.01	0.011
1–14 day	41.67 ^d^	46.29 ^c^	50.29 ^b^	52.62 ^a^	0.98	<0.01	<0.01	0.034

Abbreviations: CSB, coated sodium butyrate; 300, 300 mg/kg coated sodium butyrate; 500, 500 mg/kg coated sodium butyrate; 800, 800 mg/kg coated sodium butyrate. ^a,b,c,d^ Average with diverse superscripts in the column shows a significant difference (*p* < 0.05). Averages from 5 replicates.

**Table 5:** Effects of coated sodium butyrate on intestinal morphology of laying hens challenged with *Salmonella.*

Item	CSB Level (mg/kg)				SEM	*p*-Value		
	0	300	500	800		ANOVA	Linear	Quadratic
Duodenum								
VH (μm)	1018	1210	1264	1270	38.61	0.055	0.014	0.021
CD (μm)	172	187	177	175	4.5	0.677	0.996	0.625
VH/CD	6.05	6.45	7.13	7.23	0.19	0.065	0.008	0.029
Jejunum								
VH (μm)	899 ^b^	955 ^a^	963 ^a^	968 ^a^	9	0.009	0.004	0.004
CD (μm)	168	168	158	157	4.2	0.689	0.248	0.523
VH/CD	5.37	5.72	6.15	6.33	0.17	0.206	0.03	0.097
Ileum								
VH (μm)	478	565	606	616	23.81	0.151	0.028	0.065
CD (μm)	124	108	113	101	3.52	0.113	0.036	0.109
VH/CD	4.51	5.24	5.34	5.64	0.19	0.177	0.033	0.091

Abbreviations: CSB, coated sodium butyrate; 300, 300 mg/kg coated sodium butyrate; 500, 500 mg/kg coated sodium butyrate; 800, 800 mg/kg coated sodium butyrate. ^a,b^ Average with diverse superscripts in the column shows a significant difference (*p* < 0.05). Averages from 5 replicates. VH = villus height; CD = crypt depth; V/H = the ratio of villus height to crypt depth.

**Table 6:** Effects of coated sodium butyrate on mRNA relative expression levels of inflammatory factor-related genes in jejunum and ileum mucosa of laying hens after *Salmonella* challenge.

Item	CSB Level (mg/kg)				SEM	*p*-Value		
	0	300	500	800		ANOVA	Linear	Quadratic
Jejunum								
*IL-1β*	1.00	0.7	0.52	0.29	0.09	0.058	0.005	0.021
*IL-6*	1.00	0.63	0.65	0.52	0.08	0.142	0.034	0.084
*IL-10*	1.00	1.32	1.45	1.26	0.09	0.351	0.272	0.185
*IFN-γ*	1.00	0.74	0.68	0.85	0.08	0.536	0.529	0.325
Ileum								
*IL-1β*	1.00 ^b^	0.62 ^a^	0.50 ^a^	0.50 ^a^	0.07	0.042	0.013	0.015
*IL-6*	1.00	0.35	0.33	0.41	0.11	0.095	0.101	0.046
*IL-10*	1.00	1.31	1.63	1.85	0.14	0.125	0.013	0.05
*IFN-γ*	1.00	0.81	0.51	0.82	0.13	0.644	0.458	0.51

Abbreviations: CSB, coated sodium butyrate; 300, 300 mg/kg coated sodium butyrate; 500, 500 mg/kg coated sodium butyrate; 800, 800 mg/kg coated sodium butyrate. ^a,b^ Average with diverse superscripts in the column shows a significant difference (*p* < 0.05). Averages from 5 replicates.

**Table 7:** Effects of coated sodium butyrate on mRNA relative expression of jejunum and ileum intestinal barrier-related genes of laying hens after *Salmonella* challenge.

Item	CSB Level (mg/kg)				SEM	*p*-Value		
	0	300	500	800		ANOVA	Linear	Quadratic
Jejunum								
*ZO-1*	1.00 ^b^	1.57 ^b^	1.55 ^b^	1.86 ^a^	0.11	0.026	0.004	0.015
*CLDN1*	1.00	1.27	1.41	1.83	0.14	0.289	0.049	0.151
*MUC2*	1.00	1.26	1.5	1.29	0.07	0.084	0.087	0.044
Ileum								
*ZO-1*	1.00 ^b^	1.75 ^a^	1.66 ^a^	1.78 ^a^	0.11	0.02	0.009	0.013
*CLDN1*	1.00 ^b^	1.28 ^ab^	1.74 ^a^	1.83 ^a^	0.12	0.039	0.004	0.016
*MUC2*	1.00	1.51	1.77	1.43	0.12	0.151	0.134	0.046

Abbreviations: CSB, coated sodium butyrate; 300, 300 mg/kg coated sodium butyrate; 500, 500 mg/kg coated sodium butyrate; 800, 800 mg/kg coated sodium butyrate. ^a,b^ Average with diverse superscripts in the column shows a significant difference (*p* < 0.05). Averages from 5 replicates. CLDN1 = Claudin-1; ZO-1 = zonula occludens-1; MUC2 = Mucin-2.

**Table 8:** Effects of coated sodium butyrate on short-chain fatty acids of cecal digesta of laying hens challenged with *Salmonella.*

Item	CSB Level (mg/kg)				SEM	*p*-Value		
	0	300	500	800		ANOVA	Linear	Quadratic
Acetic acid (mmol/L)	45.84	55.13	74.3	63.92	4.68	0.164	0.079	0.121
Propionic acid (mmol/L)	15.29	20.67	26.29	24.38	1.75	0.116	0.032	0.055
Isobutyric acid (mmol/L)	1.35	1.17	1.03	1.15	0.09	0.71	0.383	0.508
Butyric acid (mmol/L)	1.18 ^b^	1.31 ^b^	1.97 ^a^	1.92 ^a^	0.13	0.04	0.008	0.03
Isovaleric acid (mmol/L)	1.92	1.63	1.59	1.61	0.07	0.321	0.124	0.174
Valeric acid (mmol/L)	1.86	2.07	2.86	2.51	0.16	0.122	0.057	0.115
T-SCFAs (mmol/L)	67.44	81.98	108.05	95.49	6.61	0.149	0.06	0.099

Abbreviations: CSB, coated sodium butyrate; 300, 300 mg/kg coated sodium butyrate; 500, 500 mg/kg coated sodium butyrate; 800, 800 mg/kg coated sodium butyrate. ^a,b^ Average with diverse superscripts in the column shows a significant difference (*p* < 0.05). Averages from 5 replicates. T-SCFAs = total short-chain fatty acid.

## Data Availability

The data presented in this study are available on request from the corresponding author. The data are not publicly available due to [the containing information that could compromise the privacy of research participants].
